# To reveal and acknowledge an iatrogenic cerebral amyloid angiopathy: a vascular neurologist and neurosurgeon collaborative perspective

**DOI:** 10.3389/fneur.2025.1527417

**Published:** 2025-06-13

**Authors:** Senta Frol, Bruno Splavski

**Affiliations:** ^1^Department of Vascular Neurology, University Medical Center Ljubljana, Ljubljana, Slovenia; ^2^Faculty of Medicine, University of Ljubljana, Ljubljana, Slovenia; ^3^Department of Neurosurgery, Dubrovnik General Hospital, Dubrovnik, Croatia; ^4^Faculty of Applied Health Sciences, University of Zagreb, Zagreb, Croatia

**Keywords:** iatrogenic cerebral amyloid angiopathy, prion-like disease, cadaveric materials transmission, previous neurosurgery procedures, perspective

## Introduction

Cerebral amyloid angiopathy (CAA) is an age-related small vessel disease characterized by the progressive deposition of amyloid-β (Aβ) in the walls of cortical and leptomeningeal blood vessels, and brain parenchyma, with advancing age being the primary risk factor for its development (1). In addition to cognitive decline, dementia, and transient focal neurological deficits, CAA can lead to spontaneous lobar intracerebral hemorrhage (ICH). Neuroradiological imaging, including brain CT and MRI, often reveals characteristic features such as cortical microbleeds, superficial siderosis, and an atypical ICH location (1, 2). Positron emission tomography (PET) is increasingly utilized to detect diffuse cortical amyloid deposits (3), while cerebrospinal fluid (CSF) analysis may demonstrate lowered Aβ levels (4). However, the definitive diagnosis requires histopathological examination of brain tissue, including samples of leptomeningeal and cortical vessels and parenchymal Aβ deposits, typically obtained through intraoperative biopsy or post-mortem autopsy (5).

Iatrogenic cerebral amyloid angiopathy (iCAA), a recently identified form of CAA, diverges from the classical age-related variant by presenting earlier in life, typically between the third and fifth decades. It is often associated with recurrent ICH, seizures, and cognitive impairment. It is thought to arise from prior exposure to cadaveric materials, such as lyophilized dural grafts, used in neurosurgical procedures, and shares pathophysiological mechanisms with prion diseases, where Aβ deposition is mediated by the transmission of misfolded proteins (6). Diagnosing iCAA requires a detailed medical history to identify potential exposure to sources of Aβ, with clinical symptoms frequently manifesting decades after the initial surgery, typically with a latency period of 30 to 40 years.

A critical analysis of current research reveals that iCAA has shifted from a theoretical concern to a documented reality with significant implications for both patient care and medical ethics. By understanding iCAA's prion-like transmission mechanism and reassessing surgical practices, the medical community can take proactive steps to reduce the incidence of this devastating, but preventable condition.

In light of these findings, this opinion paper argues for an increased clinical awareness of iCAA as a potential diagnosis in younger patients presenting with atypical ICH.

## Discussion

### The emergence of iCAA and its implications for neurosurgery

The emergence of iCAA underscores the unintended long-term effects of neurosurgical practices once thought safe. For decades, cadaveric dura mater was used as a graft material in cranial surgeries, without awareness of its potential for Aβ transmission. The latency period for iCAA, often spanning 30–40 years, creates diagnostic challenges, as symptoms appear long after initial surgical exposure, usually manifesting in patients'30 s to 50 s—an age range atypical for spontaneous CAA. This extended latency further complicates diagnosis and contributes to the under-recognition of iCAA.

The implications are far-reaching: younger patients presenting with unexplained ICH, cognitive impairment, or seizures should be evaluated for a history of neurosurgical exposure to cadaveric material, as iCAA may not respond to the same interventions effectively in age-related CAA. This demands a paradigm shift in the neurological and neurosurgical approach, where diagnostic workups should include a history of neurosurgical interventions involving cadaveric dural grafts and incorporate advanced imaging and biomarker analysis specific to Aβ accumulation.

The iCAA clues include the onset of symptoms earlier in life (<50 years), a history of neurosurgical treatment using cadaveric material, clinical and neuroradiologic features associated with CAA, evidence of Aß brain tissue, as well as the exclusion of CAA genetic causes (4). Yet, after establishing the diagnosis, long symptom-free intervals can occur, followed by multiple ICH recurrences (2).

The commonest CAA form, sporadic Aβ CAA, usually affects people in mid- to later life. However, early-onset forms, though uncommon, are being increasingly recognized and may result from genetic or iatrogenic causes that warrant specific and focused investigation and management (7). Notably, patients with iCAA are significantly younger than those with sporadic CAA, which is often related to the historical use of lyophilized dural transplants during childhood (8, 9). Therefore, the transmission of Aβ seeds from cadaveric dural grafting and/or neurosurgical instruments was suggested as a potential source of the disease in some patients (9–11). Also, a hazard for developing an iCAA and ICH after a red blood cell (RBC) transfusion cannot be overlooked (12–15).

### Prion-like transmission mechanisms and the need for clinical vigilance

One of the most troubling aspects of iCAA is its apparent prion-like transmission mechanism. Although not a prion disease in the strict sense, iCAA shares a key characteristic: the misfolding and aggregation of Aβ proteins, which appear capable of inducing pathology in host tissue. This mechanism is similar to prion diseases, which are caused by infectious proteins rather than viruses or bacteria. Prions, notably resilient against standard sterilization methods, present a formidable challenge in controlling iatrogenic transmission—a property that Aβ proteins seem to share (16).

Prion diseases, also known as transmissible spongiform encephalopathies, are caused by a fundamentally different biological mechanism of infection. These progressive neurodegenerative disorders result from prions—misfolded proteins that are the smallest infectious agents, even smaller than viruses, and lack nucleic acids (DNA or RNA) in their structure (16). Unlike other microorganisms, prions are not composed of genetic material, which makes them unique in their mode of transmission and pathogenicity. Prions are highly resistant to conventional disinfection methods, posing significant risks not only to patients but also to healthcare professionals. Transmission can occur through exposure to contaminated nervous tissue or medical instruments, including cadaveric allografts. The incubation period for prion diseases can be exceptionally prolonged, often extending over several decades before symptoms manifest.

Research has indicated Aβ transmission in patients with iatrogenic Creutzfeldt-Jakob disease (iCJD), where Aβ deposits are often present alongside prion pathology (6). This finding raises broader questions about the transmissibility of amyloid-related diseases and highlights a gap in understanding the full impact of acquired Aβ pathology, particularly in relation to iCAA. Furthermore, as evidence of amyloid transmission continues to accumulate, neurosurgical practices and sterilization protocols must adapt to address these risks. This includes reconsidering both the materials used in neurosurgery and the potential long-term impact of blood and tissue products in medical procedures. Transmission of Aβ protein has been documented in both patients with and without iatrogenic Creutzfeldt-Jakob disease (CJD) (7). However, there is limited information on the clinical implications of acquired Aβ pathology, particularly its role in the development of symptomatic CAA following childhood exposure to cadaveric dura transplants (3). The prion-like transmission of Aβ proteins through cadaveric dura, potentially occurring decades after neurosurgical procedures, has been proposed as a potential iatrogenic cause of CAA (2). Following the initial pathological description of presumed Aβ transmission in humans (17), clinical cases of iCAA have been increasingly recognized, often linked to such transmission. This newly identified form of CAA frequently presents with ICH, as well as seizures and cognitive impairment (12). A comprehensive review by Pikija et al. revealed that 82% of patients with iCAA in their cohort experienced ICH, with 11% presenting with seizures as an initial symptom (5).

### The shift toward dural synthetic materials: an ethical and practical imperative

Given the association between cadaveric dural grafts and iCAA, transitioning toward synthetic alternatives is not only advisable but also essential.

Years ago, while investigating the best dural closing after a missile penetrating brain injury, we concluded that autologous dural grafts like fascia lata of pericranium have some advantages over cadaveric transplants like lyodura, which were frequently in use these days. These advantages include more rapid wound healing due to the stimulating effect of viable tissue remaining in the autograft, which diminishes cortical scarring and probably prevents post-traumatic seizures (18). However, we never suspected that cadaveric dural transplants may cause iCAA many years later.

Dural repair after cranial and spinal neurosurgery is demanding and often presents a technical challenge. There are multiple options for dural defect repair, including autografts, allografts derived from cadaveric human or bovine lyophilized transplants, as well as recently developed entirely synthetic and absorbable dura substitutes that are not derived from biological sources, and have no risk of disease transmission (19). Accordingly, synthetic dural allografts and systematic monitoring of individuals who have had neurosurgical procedures earlier in life, especially those involving cadaveric dural transplants, are advised and required.

The potential drawbacks of using synthetic materials for dural repair include the risk of inflammatory granulomatous foreign body reactions and excessive scarring, which, in some cases, can lead to postoperative complications (20). However, we believe that the benefits of using these grafts outweigh the risk of inducing iatrogenic diseases, a concern common with other allografts.

Historically, both autologous and cadaveric grafts were used for complex dural repairs, with little consideration of long-term risks. Today, fully synthetic and biodegradable dural grafts limit the potential for disease transmission, often providing superior biocompatibility and reducing immune response. The availability of synthetic dura substitutes represents a viable and ethically sound choice for modern neurosurgery (19). Synthetic grafts are particularly attractive due to their reliable biomechanical properties and are morally acceptable because they do not raise concerns regarding medicolegal and ethical principles.

However, one should never underestimate the use of autologous grafts (like fascia lata and pericranium). They are well-vascularized, non-immunogenic, non-toxic, and non-transmissible dural substitutes. They are also readily available and cost-effective. Yet, these advantages are often shadowed by their restricted supply, ineffectiveness in large defects, and potential for infectious complications.

Therefore, from the neurosurgeons' viewpoint, the shift to synthetic grafts aligns with a commitment to not harm, mitigating a preventable risk factor for iCAA. Patients undergoing cranial surgery today should not face the long-term risk of Aβ transmission due to outdated practices and materials. Moving forward, medical institutions should encourage the exclusive use of synthetic grafts and reinforce the importance of iatrogenic disease prevention through regular reviews of surgical materials and sterilization standards. This will require broad awareness across healthcare institutions, as well as systematic tracking of individuals with prior exposure to cadaveric dura, to facilitate early detection of iCAA where possible.

Early-onset of CAA, as a small vessel disease causing spontaneous ICH, has been recently identified in patients with a history of TBI (21) or other cerebral lesions leading to neurosurgery using cadaveric dural extracts or other medical procedures like endovascular tumor embolization, many years before the first ICH event (5).

### Closing remarks

Considering the above, neurosurgeons should be aware of the potential risks of iCAA transmission while choosing the material and optimal mode of surgical management, while vascular neurologists should keep in mind the importance of early iCAA detection, prevention, and ways of treatment.

The rising recognition of iCAA represents a call for the medical community to confront the unintended consequences of past neurosurgical practices. This emerging condition challenges conventional wisdom about amyloid angiopathy, shifting our understanding of CAA from a primarily age-related disease to one with the potential for earlier, iatrogenic onset. This new perspective demands that vascular neurologists and neurosurgeons consider iCAA in differential diagnoses of younger patients presenting with atypical ICH or other neurological symptoms, especially if prior neurosurgical exposure to cadaveric materials is known. Furthermore, surveillance of patients with such histories may enable prevention, and earlier intervention, improving outcomes for those at risk.

## Conclusion

Cerebral amyloid angiopathy may be iatrogenically induced by the transmission of Aβ protein seeds, which act similarly to prions through cadaveric dura transplants. Clinicians, particularly neurosurgeons and vascular neurologists, should maintain a high index of suspicion for iCAA in younger patients presenting with spontaneous, atypically localized ICH that suggests CAA, especially when there is a history of cranial surgeries involving cadaveric transplants, such as lyophilized dura or endovascular procedures for tumor embolization. As research into this rare form of CAA progresses, our understanding of its transmission mechanisms and clinical manifestations will continue to expand, leading to improved diagnostic and therapeutic strategies.
